# Autoantibody-associated psychiatric syndromes: a systematic literature review resulting in 145 cases

**DOI:** 10.1017/S0033291720002895

**Published:** 2022-04

**Authors:** Dominique Endres, Viktoria Maier, Frank Leypoldt, Klaus-Peter Wandinger, Belinda Lennox, Thomas A. Pollak, Kathrin Nickel, Simon Maier, Bernd Feige, Katharina Domschke, Harald Prüss, Karl Bechter, Rick Dersch, Ludger Tebartz van Elst

**Affiliations:** 1 Section for Experimental Neuropsychiatry, Department of Psychiatry and Psychotherapy, Medical Center – University of Freiburg, Faculty of Medicine, University of Freiburg, Freiburg, Germany; 2 Department of Psychiatry and Psychotherapy, Medical Center – University of Freiburg, Faculty of Medicine, University of Freiburg, Freiburg, Germany; 3 Neuroimmunology Section, Institute of Clinical Chemistry, University Hospital Schleswig-Holstein Kiel/Lübeck, Kiel/Lübeck, Germany; 4 Department of Psychiatry, University of Oxford, Oxford, UK; 5 Oxford Health NHS Foundation Trust, Oxford, UK; 6 Department of Psychosis Studies, Institute of Psychiatry, Psychology and Neuroscience, King's College London, London, UK; 7 Faculty of Medicine, Center for Basics in NeuromodulationUniversity of Freiburg, Freiburg, Germany; 8 Department of Neurology and Experimental Neurology, Charité – Universitätsmedizin Berlin, Berlin, Germany; 9 German Center for Neurodegenerative Diseases (DZNE) Berlin, Berlin, Germany; 10 Clinic for Psychiatry and Psychotherapy II, Ulm University, Bezirkskrankenhaus Günzburg, Günzburg, Germany; 11 Department for Neurology, Medical Center – University of Freiburg, Faculty of Medicine, University of Freiburg, Freiburg, Germany

**Keywords:** Autoantibody, autoimmune encephalitis, autoimmune psychosis, dementia, immunological encephalopathy, schizophrenia

## Abstract

**Background:**

Autoimmune encephalitis (AE) is an important consideration during the diagnostic work-up of secondary mental disorders. Indeed, isolated psychiatric syndromes have been described in case reports of patients with underlying AE. Therefore, the authors performed a systematic literature review of published cases with AE that have predominant psychiatric/neurocognitive manifestations. The aim of this paper is to present the clinical characteristics of these patients.

**Methods:**

The authors conducted a systematic Medline search via Ovid, looking for case reports/series of AEs with antineuronal autoantibodies (Abs) against cell surface/intracellular antigens combined with predominant psychiatric/neurocognitive syndromes. The same was done for patients with Hashimoto encephalopathy/SREAT. Only patients with signs of immunological brain involvement or tumors in their diagnostic investigations or improvement under immunomodulatory drugs were included.

**Results:**

We identified 145 patients with AE mimicking predominant psychiatric/neurocognitive syndromes. Of these cases, 64% were female, and the mean age among all patients was 43.9 (±22.1) years. Most of the patients had Abs against neuronal cell surface antigens (55%), most frequently against the NMDA-receptor (*N* = 46). Amnestic/dementia-like (39%) and schizophreniform (34%) syndromes were the most frequently reported. Cerebrospinal fluid changes were found in 78%, electroencephalography abnormalities in 61%, and magnetic resonance imaging pathologies in 51% of the patients. Immunomodulatory treatment was performed in 87% of the cases, and 94% of the patients responded to treatment.

**Conclusions:**

Our findings indicate that AEs can mimic predominant psychiatric and neurocognitive disorders, such as schizophreniform psychoses or neurodegenerative dementia, and that affected patients can be treated successfully with immunomodulatory drugs.

## Introduction

Mental illnesses are common and lead to high rates of ‘years lived with disability’ (Wittchen et al., 2011). They can be divided into primary idiopathic and secondary forms with clearly identifiable organic causes (Tebartz van Elst, 2017). In the context of secondary forms, over the last two decades and following the first description of anti-N-methyl-D-aspartate (NMDA)-receptor (R) encephalitis by Dalmau et al. (2007), autoantibody (Ab)-associated autoimmune encephalitis (AE) has been increasingly recognized as playing an important role. Over the last decade, a large number of other antineuronal Abs has been identified (Dalmau & Graus, 2018; Graus et al., 2016). Most patients with AE suffer from neuropsychiatric syndromes that have clear ‘neurological signs’, such as epileptic seizures or movement disorders, and accompanying psychopathological anomalies, such as hallucinations, depressive moods, or cognitive deficits (Dalmau & Graus, 2018; Graus et al., 2016). Patients mostly show clear abnormalities in the following tests: cerebrospinal fluid (CSF) pleocytosis or the detection of isolated oligoclonal bands, epileptiform activity or localized slowing (sometimes in the form of ‘extreme delta brush’) in electroencephalography (EEG), temporomesial T2/FLAIR hyperintensities or, rarely, contrast enhancement in magnetic resonance imaging (MRI), or altered [^18^F]fluorodeoxyglucose positron emission tomography (FDG-PET) metabolism (Dalmau & Graus, 2018; Graus et al., 2016; Titulaer et al., 2013). Many patients respond well to immunomodulatory drugs and, in paraneoplastic cases, to tumor-directed treatment (Dalmau & Graus, 2018; Graus et al., 2016; Titulaer et al., 2013). AE can be distinguished by Ab type in (1) AE with Abs against cell surface antigens (NMDA-R, LGI1, etc.), which can be paraneoplastic or idiopathic, (2) AE with Abs against intracellular, onconeural antigens (Ma1/2, Ri, Hu, etc.), which are mostly paraneoplastic, (3) AE with Abs against intracellular synaptic antigens (GAD65, amphiphysin), which can be paraneoplastic (amphiphysin) or idiopathic (GAD65), and (4) non-paraneoplastic steroid-responsive encephalopathies with Abs against thyroid antigens (TPO, TG), which probably defines a heterogeneous group of syndromes (Dalmau & Graus, 2018; Endres et al., 2019a; Graus et al., 2016; Tebartz van Elst et al., 2019). Several cases and small case series have suggested that mild forms of AEs and relapses can also mimic primary psychiatric disorders, such as psychosis, without neurological symptoms. For these Ab-associated AEs with primary psychiatric syndromes, the term ‘autoimmune psychosis’ has been suggested (Ellul, Groc, Tamouza, & Leboyer, 2017; Najjar, Steiner, Najjar, & Bechter, 2018; Pollak et al., 2020).

### Rationale of the current study

The previous evidence showing that AEs can mimic psychiatric syndromes originates only from case reports or small case series (Endres et al., 2019b; 2020; Kayser, Titulaer, Gresa-Arribas, & Dalmau, 2013). To summarize previous experiences of AEs mimicking primary psychiatric syndromes regarding clinical manifestations, the typical findings in instrument-based diagnostic findings, and treatment responses, the authors conducted the present systematic literature review. The authors hypothesized that a small subgroup of AEs will present clinically as psychiatric/neurocognitive disorders and could be treated with immunomodulatory treatment. In addition, the aim of this article was to identify patterns of clinical, diagnostic, and/or treatment responses among the published cases that are associated with a particular Ab type of disease to aid in clinical diagnosis and to provide avenues for further research.

## Material and methods

### Inclusion and exclusion criteria

The authors searched for articles reporting on patients with psychiatric or neurocognitive syndromes (schizophrenia, psychotic disorders, depression, dementia, confusion, etc.) without predominant neurological signs AND the following:

- The detection of established antineuronal Abs against cell surface antigens (*NMDA-R, AMPA-1/2-R, GABA-B-R, VGKC-complex, LGI1, CASPR2, mGluR1/R5, glycine-R, DPPX, etc; including AQP4 and MOG antibodies*) OR the detection of Abs against intracellular/synaptic antigens (*GAD65, amphiphysin, Yo, Hu, Ri, Ma1/2, SOX1, AK5, Ca/ARHGAP26, etc.*) OR the detection of elevated Ab levels against thyroid antigens (*TPO, TG, TSH receptors*) AND a clinical response to any immunomodulatory therapy OR the detection of unknown antineuronal Abs (binding to brain tissue) in the CSF or serum AND
- Signs for encephalopathy/encephalitis in CSF [e.g. pleocytosis, blood–brain barrier-dysfunction, oligoclonal bands (OCBs)]/EEG (e.g. epileptic activity)/MRI (e.g. lesions)/FDG-PET (e.g. hypermetabolism) OR immunomodulatory treatment response (basic requirement for patients with antithyroid Abs) OR tumor detection OR improvement after tumor therapy.

We excluded all articles regarding patients with (1) clear neurological signs, such as focal neurological deficits or epileptic seizures, (2) autonomic dysfunction requiring medical intensive care treatment, and (3) systemic neurological/rheumatological disorders with brain involvement [such as systemic lupus erythematosus (SLE)]. Patients with common and non-specific accompanying neurological symptoms (e.g. headache) or symptoms commonly seen in primary idiopathic psychiatric disorders and which could also be because of psychotropic drugs (e.g. rigidity, tremor, slight reduction of consciousness) were not excluded.

For this approach, the clinical perspective of the psychiatrist in the emergency service was chosen.

### Literature research

**Fig. 1.**
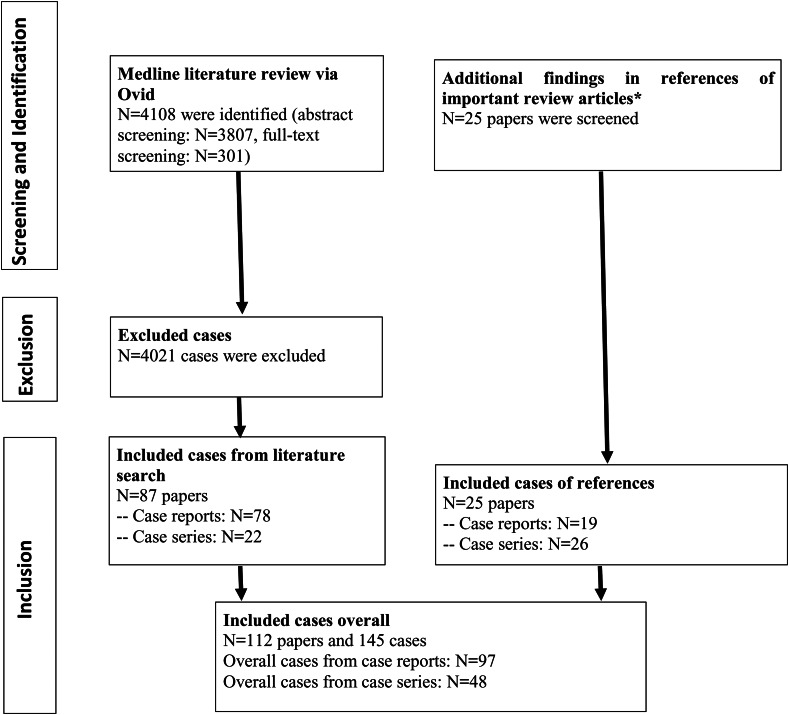
PRISMA flow diagram. Abbreviations: CS, case series; CR, case report. *References of screened reviews: (Al-Diwani, Pollak, Langford, & Lennox, 2017a; Al-Diwani, Pollak, Irani, & Lennox, 2017b; Bien & Bauer, 2013; Castillo et al., 2006; Chong et al., 2003; Dalmau, 2016; Dalmau et al., 2011; Dalmau, Geis, & Graus 2017; Dalmau & Rosenfeld, 2014; Dalmau & Vincent, 2017; Ehrenreich, 2017; Graus et al., 2016; Herken & Prüss, 2017; Kayser, Kohler, & Dalmau 2010; Kayser & Dalmau, 2011a, 2011b, 2016; Lancaster, 2016; Laurent et al., 2016; Lewerenz, Jarius, Wildemann, Wandinger, & Leypoldt, 2016; Leypoldt, Armangue, Dalmau, 2015; Menon et al., 2017; Najjar & Pearlman, 2015; Prüss & Lennox, 2016; Tebartz van Elst, Stich, & Endres, 2015; Titulaer & Dalmau, 2014; Zandi, Lennox, & Vincent, 2016; Zuliani, Graus, Giometto, Bien, & Vincent, 2012).

We conducted a Medline literature search in the database Ovid (date of search 17.10.2018). The detailed search strategy is shown in online Supplementary Table S1. The literature search resulted in 4108 hits. The discovered sources were screened for matching cases in the case reports and case series by a trained expert rater (VM). First, an abstract screening was performed for all articles and then a full-text analysis for suspicious cases. Moreover, the references of important reviews were screened for additional eligible references (for these references, see Fig. 1). All query cases were reevaluated in a second step by a board-certified psychiatrist. Finally, 145 cases met our inclusion criteria and could be included in the review (see Fig. 1).

### Subgroup definition

We classified patients by Ab type: (1) against neuronal cell surface antigens, (2) against intracellular/synaptic antigens, and (3) with steroid-responsive encephalopathy associated with autoimmune thyroiditis (SREAT)/Hashimoto encephalopathy. We also distinguished patients by predominant clinical syndrome (schizophreniform, amnestic/dementia-like, depressive, manic, confusional, anxiety-compulsive, tic, sleep disturbance, or autistic). The schizophreniform group included patients with paranoid-hallucinatory, catatonic, hebephrenic, and schizoaffective syndromes. The confusional syndrome category represented a residual category that included patients with confusion and other accompanying symptoms that could not be assigned to the other syndrome groups. The severity of brain involvement was assessed based on the number of additional abnormal findings from MRIs, EEGs, and CSFs.

### Statistics

The data were transferred to a Statistical Package for the Social Sciences, version 24 (IBM, New York, USA) database. The main results are presented descriptively. The subgroup comparisons (between patients with different Abs) for categorical variables (e.g. gender, therapy response) were carried out using Pearson's χ^2^ test or via binominal logistic regression for age-dependent variables (e.g. CSF, MRI, or EEG pathologies). The dimensional variables (e.g. age) were compared using a one-way ANOVA. For all statistical analyses, a *p* value of <0.05 served as the criterion of significance.

## Results

The detected cases were published between 1999 and October 2018. The year with the greatest number of publications was 2015 (*N* = 25; 17%). Most publications originated from European (*N* = 64; 44%) and Asian authors (*N* = 44; 30%). In addition, 36 cases were published by American authors (24%) and one case (1%) by an African author.

### Sociodemographic findings

The sociodemographic data are summarized in Table 1. Twenty-five patients (17%) were under 18 years old, and 29 patients were over 65 years old (20%). The analyses of the age distribution for 10-year intervals showed two peaks: the largest peak was in the ages between 10 and 19 years (*N* = 26; 18%), and the second largest peak was between 60 and 69 years (*N* = 25; 17%).

**Table 1.** Antibody findings and syndromes

	All patients	Abs against cell surface antigens^a^	Abs against intracellular antigens	Hashimoto encephalopathy/SREAT
Number of cases (% per subgroup)	145 (100%)	80 (55%)	15 (10%)	50 (34%)
Females (%)	93 (64%)	49 (61%)	9 (60%)	35 (70%)
Males (%)	52 (36%)	31 (39%)	6 (40%)	15 (30%)
Age ± s.d. in years (range)	43.9 ± 22.1 (from 2–91 years)	40.7 ± 22.1 (from 2–91 years)	56.9 ± 20.6 (from 19–85 years)	45.1 ± 21.2 (from 10–84 years)
Antibody subtypes (% per subgroup)	Abs against cell surface antigens: 80 (55%) Abs against intracellular antigens: 15 (10%) Hashimoto encephalopathy: 50 (34%)	Anti-NMDA-R: 46 (58%) Anti-VGKC: 18 (23%) LGI1: 4 (5%) CASPR2: 0 (0%) Anti-AMPA: 12 (15%) Anti-GABA_A_: 1 (1%) Anti-DPPX: 1 (1%) Anti-AQP4: 1 (1%) Unknown: 1 (1%)	Anti-GAD65: 1 (7%) Anti-Ca/ARHGAP26 ab: 1 (7%) Anti-Ma1/2: 3 (20%) Anti-Ri: 2 (13%) Anti-Hu: 1 (7%) Anti-Yo: 1 (7%) Anti-AK5: 5 (33%) Anti-BRSK2: 1 (7%)	Anti-TPO: 22 (44%) Anti-TG: 2 (4%) Anti-TPO + TG: 25 (50%) Unclear: 1 (2%) (‘antithyroid antibody’)
Antibody investigation in serum/CSF	Serum: 135 (93%) CSF: 69 (48%)	Serum: 70 (88%) CSF: 47 (59%)	Serum: 15 (100%) CSF: 8 (53%)	Serum: 50 (100%) CSF: 14 (28%)
Antibody detection in serum/CSF	Serum: 119 from 135 (88%) CSF: 52 from 69 (75%) Unclear: 6	Serum: 57 from 70 (81%) CSF: 38 from 47 (81%) Unclear: 6	Serum: 12 from 15 (80%) CSF: 8 from 8 (100%) Unclear: 0	Serum: 50 from 50 (100%) CSF: 6 from 14 (43%) Unclear: 0
Syndromes (% per subgroup)				
Schizophreniform	50 (34%)	29 (36%)	3 (20%)	18 (36%)
Amnestic/dementia-like	57 (39%)	28 (35%)	12 (80%)	17 (34%)
Confusional	16 (11%)	11 (14%)	0 (0%)	5 (10%)
Depressive	11 (8%)	5 (6%)	0 (0%)	6 (12%)
Manic	5 (3%)	3 (4%)	0 (0%)	2 (4%)
Tic	1 (1%)	0 (0%)	0 (0%)	1 (2%)
Sleep-disturbance	2 (1%)	2 (3%)	0 (0%)	0 (0%)
Autistic	2 (1%)	2 (3%)	0 (0%)	0 (0%)
Anxiety-compulsive	1 (1%)	0 (0%)	0 (0%)	1 (2%)

SD, standard deviation; CSF, cerebrospinal fluid; SREAT, steroid-responsive encephalopathy associated with autoimmune thyroiditis.

More than one antibody (Ab) could have been positive in individual cases, in these cases, only the predominant Ab was mentioned here. From the anti-VGKC Ab positive patients, seven cases were screened for anti-LGI1 and five cases were screened for anti-CASPR2 subtype. One patient with intracellular Abs also had anti-VGKC-Abs. Five patients with antineuronal Abs against cell surface, intracellular Abs and anti-thyroid Abs also had other intracellular Abs. Five patients with antineuronal Abs against cell surface and intracellular antigens also had reported anti-thyroid Abs.

a Patients with anti-AQP4 Abs were added to the group of patients with Abs against cell surface antigens for subgroup analyses.

### Antibody types

In total, 80 of the patients had Abs against cell surface antigens (55%), 15 had Abs against intracellular antigens (10%), and 50 patients were classified as SREAT/Hashimoto encephalopathy (34%). Anti-NMDA-R Abs (*N* = 46) and anti-VGKC-complex Abs (*N* = 18; four of them were also positive for LGI1) were the most frequent antineuronal Abs. Patients with anti-TPO (without or with TG) Abs and a steroid response (*N* = 47) were even more frequent. The Ab distribution is presented in detail in Table 1. Overall, CSF Ab screening was performed only in 69 cases and was positive in 75% of these. For most patients, the Ab isotype was not mentioned. However, most of those that were mentioned (22 of 24) were immunoglobulin (Ig) G Abs (in one case combined with IgM and IgA), isolated IgM Abs were described for two patients. Information about the Ab testing method was available for 52 cases (36%). Anti-NMDA-R Ab levels were often not reported. Anti-VGKC serum Ab concentrations (radio-immuno-assay) were reported in 15 of the 20 cases; they varied between 102 and 2680 pmol/l (mean value: 828 pmol/l). In the group of SREAT/Hashimoto encephalopathy, the results of antineuronal Abs search were reported explicitly in only 16 out of 50 patients (32%). One of these cases had additional anti-Yo Abs.

### Clinical syndromes

Most patients suffered from amnestic/dementia-like (*N* = 57; 39%), schizophreniform (*N* = 50; 34%), and confusional syndromes (*N* = 16; 11%) (see Table 1).

### Clinical symptoms

Memory dysfunctions were the most common impairment and described in 84 case reports (58%). Energy and psychomotor dysfunctions were reported in 73 (50%), affective symptoms in 55 (38%), concentration and attention deficits in 51 (35%), sleep disturbance in 44 (30%), formal thought disorder in 43 (30%), auditory hallucinations in 37 (26%), delusions in 32 (23%), orientation problems in 31 (21%), optical perception dysfunction in 24 (17%), and fear/obsessive-compulsive symptoms in 14 (10%) cases. Suicidality was described for 10 patients (7%), and relevant aggression was reported for a different group of 17 patients (12%). Accompanying autonomic dysregulation was mentioned in 17 case reports (12%); most frequently, urinary incontinence (*N* = 5; 3%), fever/diaphoresis (*N* = 4; 3%), tachycardia (*N* = 3; 2%), and arterial hypertension (*N* = 3; 2%) were reported. Accompanying neurological symptoms were found in 46 cases (32%). The following neurological symptoms – which did not dominate the clinical picture according to the case reports – were found: motor symptoms (e.g. rigor or tremor; *N* = 22; 15%), headache (*N* = 12; 8%), disturbances/fluctuations of consciousness (*N* = 7; 5%), speech disorder (e.g. dysarthria; *N* = 4; 3%), myoclonic jerks (*N* = 2; 1%), sensory disturbances (*N* = 2; 1%). In four patients, epileptic grand mal/focal seizures were discussed but not proven (3%). Neurological symptoms were most frequently found in patients with Abs against cell surface antigens (*N* = 29; 36%) and in patients with Hashimoto encephalopathy (*N* = 15; 30%); and infrequent in patients with Abs against intracellular antigens (*N* = 2; 13%; *p* = 0.205).

### Diagnostic findings and tumor association

The diagnostic work-up that most frequently showed abnormalities was CSF analysis (in 78% of the reported cases). EEGs revealed alterations in 61% of the cases, and MRI pathologies were described in 51% of the cases. When analyzing the results of these three main clinical diagnostic procedures (CSF analyses, EEG, MRI), only one abnormality was found in 44 cases (30%), two alterations in 57 cases (39%), and three pathologies in 22 case reports (15%). FDG-PET results of the brain were reported for nine cases (seven patients had Abs against cell surface antigens) and alterations were detected in all patients (100%); predominant hypermetabolic alterations were found in five (56%) and hypometabolic changes in four cases (44%). Tumor detection was described in 32 cases (22%). None of the patients with Hashimoto encephalopathy had an associated neoplasm (Table 2).

**Table 2.** Findings of instrument-based diagnostics

	All patients (*N* = 145)	Patients with Abs against cell surface antigens (*N* = 80)	Patients with Abs against intracellular antigens (*N* = 15)	Hashimoto encephalopathy/SREAT (*N* = 50)
CSF analyses				
CSF analyses/findings reported	126 (87%)	72 (90%)	14 (93%)	40 (80%)
CSF overall alterations (% of cases with reported CSF results)	98 (78%)	64 (89%)	11 (79%)	23 (58%)
Increased WBC count (explicit reported res.; altered % of them)	43 (of 103; 42%)	32 (of 59; 54%)	5 (of 12; 42%)	6 (of 32; 19%)
Increased protein concentration/albumin quotient (explicit reported res.; altered % of them)	38 (of 101; 38%)	19 (of 54; 35%)	7 (of 13; 54%)	12 (of 34; 35%)
Oligoclonal bands in the CSF (explicit reported res.; altered % of them)	18 (of 118; 15%)	12 (of 67; 18%)	4 (of 13; 31%)	2 (of 38; 5%)
Instrument-based diagnostics				
EEG investigations reported	98 (68%)	55 (69%)	6 (40%)	37 (74%)
EEG overall alterations (% of cases with reported EEG results)	60 (61%)	35 (64%)	3 (50%)	22 (59%)
EEG slowing (% of cases with reported EEG findings)^a^	48 (50%)	25 (45%)	3 (50%)	20 (54%)
Epileptic activity (% of cases with reported EEG findings)^a^	11 (11%)	9 (16%)	0 (0%)	2 (5%)
Extreme delta brush (% of cases with reported EEG findings)	1 (1%)	1 (2%)	0 (0%)	0 (0%)
MRI results reported	134 (92%)	76 (95%)	14 (93%)	44 (88%)
MRI overall alterations (% of cases with reported MRI results)	69 (51%)	41 (54%)	12 (86%)	16 (36%)
Limbic lesions^b^	34 (25%)	24 (32%)	9 (64%)	1 (2%)
Extra-limbic lesions^b^	21 (16%)	13 (17%)	1 (7%)	7 (16%)
Cortical generalized atrophy^b^	7 (5%)	1 (1%)	1 (7%)	5 (11%)
Localized cortical atrophy^b^	4 (3%)	1 (1%)	1 (7%)	2 (5%)
Post-ischemic defects^b^	3 (2%)	2 (3%)	0 (0%)	1 (2%)
Tumor association overall (% per full group)	32 (22%)	25 (31%)	7 (47%)	0 (0%)
Ovarian-Ca	10 (7%)	9 (11%)	1 (7%)	0 (0%)
Bronchial-Ca	7 (5%)	4 (5%)	3 (20%)	0 (0%)
Mamma-Ca	4 (3%)	3 (4%)	1 (7%)	0 (0%)
Thymoma	3 (2%)	2 (3%)	1 (7%)	0 (0%)
Hematological neoplasia	3 (2%)	3 (4%)	0 (0%)	0 (0%)
Gastrointestinal tumor	3 (2%)	2 (33%)	1 (7%)	0 (0%)
Other^c^	2 (1%)	2 (3%)	0 (0%)	0 (0%)

CSF, cerebrospinal fluid; EEG, electroencephalography, MRI, magnetic resonance imaging, CA; carcinoma; SREAT, steroid-responsive encephalopathy associated with autoimmune thyroiditis.

a If slowing and spikes were described, only spikes were coded.

b Only the predominant MRI alteration was coded.

c Others: cervical carcinoma, seminoma.

### Immunomodulatory treatment approaches

Immunomodulatory treatment was performed in 126 cases (87%). A total of 118 cases (94%) benefited from these interventions as reported by the primary authors. Seventy-one patients (56%) were treated with only one immunomodulatory drug, and 69 of these patients (97%) benefited from it. Fifty-five patients (44%) were treated with more than one immunomodulatory drug, and improvement was reported for 49 cases (89%). Overall, most patients were treated with steroids (*N* = 119; 94%) and 106 subjects (89%) improved. Most frequently, intravenous immunoglobulins (*N* = 51, 43%) and plasma exchange (*N* = 42, 33%) were used as second- and third-line interventions. The detailed therapy approaches and responses are summarized in Table 3.

**Table 3.** Immunomodulatory treatment response

	All patients (*N* = 145)	Patients with Abs against cell surface antigens (*N* = 80)	Patients with Abs against intracellular antigens (*N* = 15)	Hashimoto encephalopathy/SREAT (*N* = 50)
Immunomodulatory treatment in general^a^	126 (87%)	70 (88%)	6 (40%)	50 (100%)
Monotherapy	71 (56%)	26 (37%)	1 (17%)	44 (88%)
Polytherapy	55 (44%)	44 (63%)	5 (83%)	6 (12%)
Steroids	119 (94%)	65 (93%)	4 (67%)	50 (100%)
High-dose/low dose/unknown^b^	71 (60%)/30 (25%)/18 (15%)	40 (62%)/9 (14%)/16 (25%)	1 (25%)/3 (75%)/0 (0%)	30 (60%)/18 (36%)/2 (4%)
Monotherapy	68 (57%)	24 (40)	0 (0%)	44 (88%)
Polytherapy	51 (43%)	41 (63%)	4 (100%)	6 (12%)
IVIGs	42 (33%)	34 (49%)	6 (100%)	2 (4%)
Plasma exchange	16 (13%)	13 (19%)	1 (17%)	2 (4%)
Rituximab	6 (5%)	4 (6%)	0 (0%)	2 (4%)
��Cyclophosphamide	6 (5%)	4 (6%)	1 (17%)	1 (2%)
Others	10 (8%)	7 (10%)	1 (17%)	2 (4%)
Improvement through immunomodulatory drugs in general	118 (94%)	64 (91%)	4 (67%)	50 (100%)
Monotherapy/polytherapy	69 (58%)/49 (42%)	24 (38%)/40 (63%)	1 (25%)/3 (75%)	44 (88%)/6 (12%)
Improvement through steroids	106 (89%)	55 (85%)	3 (75%)	48 (96%)
High-dose/low-dose/unknown^b^	61 (58%), 28 (26%), 17 (16%)	32 (58%), 8 (15%), 15 (27%)	1 (33%), 2 (67%), 0 (0%)	28 (58%), 18 (38%), 2 (4%)
Monotherapy	66 (62%)	22 (40%)	0 (0%)	44 (92%)
High-dose/low-dose/unknown	38 (58%)/21 (32%)/7 (11%)	13 (59%)/4(18%)/5 (23%)	0 (0%)/0 (0%)/0 (0%)	25 (57%)/17 (39%)/2 (5%)
Polytherapy	40 (38%)	33 (60%)	3 (100%)	4 (8%)
High-dose/low-dose/unknown	23 (58%)/7 (18%)/10 (25%)	19 (58%)/4 (12%)/10 (30%)	1 (33%)/2 (67%)/0 (0%)	3 (75%)/1 (25%)/0 (0%)
Improvement through IVIGs	33 (79%)	28 (82%)	4 (67%)	1 (50%)
Monotherapy/polytherapy	4 (12%)/29 (88%)	3 (11%)/25 (89%)	1 (25%)/3 (75%)	0 (0%)/1 (100%)
Improvement through plasma exchange	14 (88%)	11 (85%)	1 (100%)	2 (100%)
Monotherapy/polytherapy	0 (0%)/14 (100%)	0 (0%)/11 (100%)	0 (0%)/1 (100%)	0 (0%)/2 (100%)
Improvement through rituximab	5 (83%)	3 (75%)	0 (0%)	2 (100%)
Monotherapy/polytherapy	0 (0%)/5 (100%)	0 (0%)/3 (100%)	0 (0%)/0 (0%)	0 (0%)/2 (100%)
Improvement through cyclophosphamide	4 (67%)	2 (50%)	1 (100%)	1 (100%)
Monotherapy/polytherapy	0 (0%)/4 (100%)	0 (0%)/2 (100%)	0 (0%)/1 (100%)	0 (0%)/1 (100%)
Psychopharmacological treatment				
Antipsychotics	46 (32%)	24 (30%)	3 (20%)	19 (38%)
Anticonvulsants	35 (24%)	22 (28%)	1 (7%)	12 (24%)
Antidepressants	20 (14%)	10 (13%)	0 (0%)	10 (20%)

IVIGs, intravenous immunoglobulins; SREAT, steroid-responsive encephalopathy associated with autoimmune thyroiditis.

a For two cases, immunomodulatory treatment was documented but without information about the outcome; we considered these cases as non-responders.

b ‘High-dose’ steroids were defined as steroid doses >500 mg, doses <500 mg were already defined as ‘low-dose’.

### Subgroup analyses

In the next step, we analyzed the differences between the subgroups of patients with (1) Abs against neuronal cell surface antigens, (2) Abs against intracellular antigens, and (3) Hashimoto encephalopathy/SREAT. The descriptive findings are summarized in Tables 2 and 3.

- **Age and gender:** The subgroups differed significantly in age (*F* = 3.64; *p* = 0.029). Patients with Abs against intracellular antigens were the oldest, followed by patients with Hashimoto encephalopathy. There were no significant differences in the gender distribution between the three subgroups (χ^2^= 1.15; *p* = 0.563).
- **Clinical syndromes:** The clinical syndromes showed no significantly different distribution (χ^2^ = 20.55; *p* = 0.196). Patients with Abs against cell surface antigens were most commonly afflicted by schizophreniform syndromes (36%), patients with Abs against intracellular antigens by amnestic/dementia-like syndromes (80%), and patients with Hashimoto encephalopathy by schizophreniform syndromes (36%).
- **Diagnostic investigations:**
  - o **EEG:** The frequency of EEG pathologies did not differ between the three subgroups (*W* = 0.93; *p* = 0.627).
  - o **CSF:** CSF pathologies were found in significantly different frequencies between the subgroups (*W* = 15.41; *p* < 0.001). The most common CSF abnormalities were seen in patients with Abs against cell surface antigens (in 89%).
  - o **MRI:** Significant differences were observed in the occurrence of MRI changes (*W* = 8.66; *p* = 0.013). Patients with Abs against intracellular antigens most often displayed MRI pathologies (86%).
  - o **Tumor association:** There was a significant difference in the presence of accompanying tumor diseases (χ^2^ = 23.36; *p* < 0.001). Tumors were detected in 47% of the patients with Abs against intracellular antigens and in 31% of the patients with Abs against cell surface antigens and in no patients with Hashimoto encephalopathy/SREAT.
- **Immunomodulatory treatment response:** All patients with Hashimoto encephalopathy/SREAT responded to immunomodulatory treatment (inclusion criterion). The comparison between patients of the two other groups showed a trend to better responses to immunomodulatory drugs in patients with Abs against cell surface antigens (χ^2^ = 3.60; *p* = 0.058).

In online Supplementary Table S2, we describe the differences between patients with different established Abs: Patients with anti-NMDA-R Abs were relatively young (29.45 ± 16.41 years) and typically suffered from schizophreniform symptoms (in 57%). Patients with anti-VGKC Abs were older (53.22 ± 24.39 years) and typically suffered from amnestic/dementia-like syndromes (72%). In 91% of the patients who were investigated for anti-NMDA-R Abs in the CSF, the samples were Ab positive. In online Supplementary Table S3, we present the findings of patients with exclusively psychiatric/neurocognitive syndromes (*N* = 99); these patients mostly suffered from anti-NMDA-R, anti-thyroid, and anti-VGKC Abs. CSF alterations were reported in 78%, EEG pathologies in 61%, and MRI alterations in 55% of the cases. Improvement through immunomodulatory medication was reported in 93% of the cases.

## Discussion

This systematic literature review, from which we identified 145 relevant cases, supports the hypothesis that AE can manifest with predominant psychiatric or neurocognitive syndromes. Importantly, most of the patients who were treated with immunomodulators benefited (94%). Our findings illustrate that AEs could have clinical significance for general psychiatry because they may mimic idiopathic psychiatric/neurocognitive disorders, which could have therapeutic consequences.

### Sociodemographic characteristics

Females were affected more often by AEs, which is in line with the observations of other autoimmune disorders (Riemekasten & Siegert, 2014). Stronger adaptive immune responses in women could lead to better control of infections and *vice versa* to an increased risk of autoimmune diseases. Immunological explanation mechanisms are complex and include hormonal, genetic, and epigenetic effects as well as possibly a sex bias in autoimmunity due to different responses to injuries (Gold, Willing, Leypoldt, Paul, & Friese, 2019). The mean age of incidence was in the mid-40s, but the two largest peaks in the course of the first presentations were found in the age group of 10–19 years and 60–69 years. The first peak in adolescence illustrates the importance of this differential diagnosis in childhood and adolescent psychiatric settings. Patients under the age of 18 most often suffered from syndromes associated with Abs against cell surface antigens; the studied patients did not have Abs against intracellular antigens. In several young girls with anti-NMDA-R Abs, ovarian teratomas could be detected. Interestingly, there were also two young patients (2 and 3 years old) with autistic regression, anti-NMDA-R Abs, and inflammatory CSF syndromes, and they showed an improvement under immunomodulatory treatment. The same applies to a 12-year-old patient with a tic disorder with antithyroid Abs, who was successfully treated with steroids (Saygi, Ozkale, & Erol, 2014; Scott et al., 2014). These three cases show that suspected developmental disorders may also be mimicked by autoimmune and thereby treatable causes. This observation is important in the context of the discussion of the possible causes of autistic regression and its putative relationship to phenomena such as the Landau–Kleffner syndrome (Tebartz van Elst & Perlov, 2013). The second peak was at an advanced age (60–69 years). Paraneoplastic processes are of particular interest here. In the current study, patients with paraneoplastic Abs against intracellular antigens were significantly older and most frequently had amnestic/dementia-like syndromes.

### Antibody types and clinical syndromes

Overall, anti-NMDA-R, antithyroid (TG, TPO), and anti-VGKC-complex Abs were most frequently found. Anti-NMDA-R Glu-NR1 Abs are associated with anti-NMDA-R encephalitis. In the initial stage, classical psychotic symptoms usually occur. Over the course of the disease, clear neurological symptoms, such as epileptic seizures, dyskinesias, increased muscle tone, dystonic body posture, or reduced consciousness, can arise (Dalmau et al., 2019; Dalmau & Graus, 2018; Graus et al., 2016). It is possible that the patients included here were detected early enough before neurological symptoms could occur, but it is also conceivable that ‘milder’ forms may lead to primarily psychiatric symptoms only. Interestingly, five patients had anti-NMDA-R Glu-NR2 Abs, which have been associated with neuropsychiatric SLE (Wang, Zhao, Zhang, & Lei, 2019). In this study, a positive anti-LGI1 status was reported in four patients. Although epileptic seizures are one of the diagnostic criteria of limbic encephalitis (Graus et al., 2016), none were observed in the cases described in this review; however, this is not surprising since seizures in anti-LGI1 encephalitis can sometimes escape detection and a clear neurological presentation of a case would have led to exclusion from the study since we were looking at primary psychiatric/neurocognitive presentations of AE. No patients with anti-CASPR2 Abs – typically associated with variable peripheral CNS symptoms, such as Morvan syndrome (van Sonderen, Schreurs, Wirtz, Sillevis Smitt, & Titulaer, 2016b) – were found. The clinical relevance of anti-VGKC complex (VGKCC) Abs without LGI1 or CASPR2 antibodies is considerably smaller (Lang et al., 2017; van Sonderen et al., 2016a). In these patients, a heterogeneous spectrum of neuropsychiatric syndromes (Prüss & Lennox, 2016; van Sonderen et al., 2016b) and a limited response to immunomodulatory drugs has been observed (Lang et al., 2017); their pathophysiological relevance is thus increasingly viewed critically (Lang et al., 2017; van Sonderen et al., 2016a). Nevertheless, we included cases, in whom only anti-VGKCC Abs were determined because testing for LGI1 and CASPR2 abs was not available before 2010 (Lang et al., 2017; Prüss & Lennox, 2016; van Sonderen et al., 2016a, 2016b). This could have led to the inclusion of patients with unspecific VGKCC Ab findings. However, the high anti-VGKCC Ab titers with an average of 828 pmol/l are comparable with the concentrations usually observed in anti-LGI1 encephalitis case series (van Sonderen et al., 2016c) and higher than in patients with anti-VGKCC Abs without LGI1 or CASPR2 abs (van Sonderen et al., 2016b). This finding and the fact that 88% of the immunomodulatory treated patients with anti-VGKC Abs benefited from it rather make a case, in the authors’ view, against non-specific findings. Even though it is rare thyroid Abs still maybe associated with Hashimoto encephalopathy. A remarkably large number of cases with primarily psychiatric symptoms were found in this paper contrasting to previous reviews that rather pointed to a mixed neuropsychiatric phenotype with aphasia, ataxia, stroke-like episodes, or epileptic seizures (Castillo et al., 2006; Chong, Rowland, & Utiger, 2003; Laurent et al., 2016). A recently published review also highlighted the role of Hashimoto encephalopathy in psychiatry (Menon, Subramanian, & Thamizh, 2017). However, many experts also take a critical view of this and point out that these cases could also conceal previously unknown antineuronal Abs (Endres et al., 2019a). The current paper supports this critical view in that only in 32% of the cases with Hashimoto encephalopathy, a search for antineuronal Abs was described explicitly. In addition, in cases where established antineuronal Abs were excluded, for example, by standard serum testing, new, still unknown and therefore undetected antineuronal Abs may have been present. To exclude these Abs, tissue-based assays on (un)fixed brain sections would be favorable (Tebartz van Elst et al., 2019). However, irrespective of the exact pathophysiology, the findings illustrate that thyroid Abs, when combined with other conspicuous, additional findings (especially in CSF and EEG), may point to underlying autoimmune pathomechanisms in some cases.

Syndrome classification was often difficult because mixed psychiatric syndromes were described. This is also highlighted by the fact that many patients were assigned to the residual category of confusional syndromes. According to current expert opinion and in accordance with our findings, an autoimmune genesis should be considered, especially in the case of atypical symptoms such as rapid onset, bizarre clinical presentation with mixed symptoms including neurological symptoms and signs (Herken & Prüss, 2017).

### Diagnostic and therapeutic consequences

The diagnostic procedures most frequently revealed CSF abnormalities (78%) in these cases. A total of 42% showed pleocytosis in the CSF, and 15% displayed OCBs. Both findings are clear signs of an inflammatory intrathecal process and should always be taken as strong signs warranting further organic clarification, including Ab testing in the clinical routine. The EEG was the second most sensitive method in detecting brain involvement. However, EEG results were only described in 68% of the patients. In our sample, the EEG phenomenon of ‘extreme delta brush’, which is considered specific for anti-NMDA-R encephalitis and which was found in severely ill patients with a Glasgow Coma Scale score <8 (Schmitt et al., 2012), was only described in one patient. It is possible that the phenomenon is particularly found in critically ill patients with classical anti-NMDA-R encephalitis. The MRI was abnormal in only half of the cases and hence seems to be less sensitive in indicating immunological pathomechanisms – as equally shown in large studies on anti-NMDA-R encephalitis (here only in 33% of the patients’ pathological MRIs were detected; Titulaer et al., 2013). Therefore, an unremarkable MRI scan is definitely not sufficient to rule out an autoimmune genesis of a psychiatric syndrome. Tumors were found in 22% of the cases. As expected, tumor association was most frequently found in patients with paraneoplastic Abs against intracellular antigens. However, only in half of these patients, there was a tumor detectable. It remains unclear whether the patients without a tumor eventually developed tumors over the course of the disease and whether the psychiatric syndrome might be an early paraneoplastic warning sign of such.

Of the patients treated with immunosuppressive therapy, most (94%) responded to this treatment. However, this finding is subject to a relevant publication bias because the patients responding to therapy are preferentially published, and additionally, a successful response to immunomodulatory drugs was required for a diagnosis of Hashimoto encephalopathy to be made, which was an inclusion criterion for the current study. Still, 91% of patients with Abs against cell surface antigens also responded to immunomodulatory treatment. The good therapeutic response of this group is probably due to the direct pathophysiological role of Abs against cell surface antigens. Regarding the most frequently detectable anti-NMDA-R Abs, it has been shown that they are directly responsible for the pathophysiological processes (Kreye et al., 2016). The Abs crosslink to the NMDA receptors changes the interaction with other synaptic proteins and causes reversible internalization of the receptors (Dalmau et al., 2019). This leads to electrochemical, electrophysiological, and neuroplastic changes (Dalmau et al., 2019; Kreye et al., 2016). Patients most frequently benefited from steroids; however, this finding is likely biased because the patients who were successfully treated with steroids did not need any further immunosuppressive treatment. Only 67% of the patients with Abs against intracellular antigens benefited from immunosuppressive treatment. This poor response can be explained pathophysiologically for this subgroup because a misdirected response of cytotoxic T cells is assumed to be the cause of the resulting inflammatory nerve damage, which is often irreversible; hence, there is often a defect state prior to starting immunomodulatory treatment (Stich & Rauer, 2014).

### Limitations

The retrospective case series approach of the present paper clearly has some limitations: the findings were not recorded uniformly and were evaluated by different investigators who used different methods. For example, there are large methodological differences in Ab testing (Jézéquel et al., 2017). In many cases, no information was provided about the methodological aspects of Ab testing, or the Ab levels were not reported. A further shortcoming is that often, no Ab tests were performed in CSF, which should be standard today – at least for the Abs against cell surface antigens. Despite all the limitations, most patients responded to immunomodulatory therapy, making it unlikely that the included cases had only non-specific Abs. There also might be a relevant publication bias because the cases that responded to therapy were probably more likely to be published than unsuccessful ones. Antibody-negative cases of AE were not detected by our searching strategy (Najjar et al., 2018). The syndrome assignment was based on the predominant syndrome relying on the descriptions found in the case reports. However, it must be stressed that frequently mixed clinical pictures were found, so the assignment was difficult and not always clear. There are symptomatic overlaps with neurological disorders, particularly when it comes to cognitive phenomena such as attention, memory, and speech. For the current paper, the clinical perspective of the psychiatrist in the emergency service was chosen. Therefore, confusion syndromes without seizures/paralysis or severe autonomic dysfunction were also included because these patients are often treated in psychiatric departments, and respective non-specific neurocognitive symptoms are frequently classified as part of an assumed schizophreniform or otherwise psychotic syndrome, particularly when substance abuse or psychotropic medication is given. The same approach was chosen with respect to non-specific neurological symptoms, such as a headache, that could not be clearly attributed to the autoimmune syndrome. However, patients with clear neurological symptoms (e.g. epileptic seizures) were consistently excluded. In the authors’ opinion, this procedure provides the highest ecological validity. Notably, this work gives only a broad first systematic overview. Future studies should focus on more details of the psychiatric manifestations of AE (e.g. individual Ab types or syndromes and comparison with patients suffering from full-blown AE).

## Conclusion

In the current systematic review focusing on patients with probable autoimmune-mediated psychiatric disorders, a relevant group of patients with a generally positive response to immunomodulatory drugs was found. Although this systematic collection of literature cases has many limitations, especially with regard to a relevant publication bias, it shows that Ab-associated AE presenting as primary psychiatric syndromes could play a role in the clinical setting of general psychiatry. Anti-NMDA-R Abs were the most common antineuronal Abs, but the spectrum of Abs was broad overall. Patients with Abs against cell surface antigens were the youngest and suffered mostly from schizophreniform symptoms. Patients with Abs against intracellular antigens were the oldest and suffered most frequently from amnesic/dementia-like syndromes. Patients with Hashimoto's encephalopathy were (on average) between the other two groups regarding age, and most patients suffered from schizophreniform or amnesic/dementia-like syndromes. A sufficient exclusion of other autoimmune causes was often not performed in patients with Hashimoto encephalopathy, supporting the idea of a poorly defined syndrome. Overall, CSF abnormalities were detected most frequently during diagnostic work ups. Further studies in this field are needed to better understand the spectrum of ‘autoimmune psychiatric syndromes’ and thereby develop optimized diagnostic and therapeutic strategies.

## Data Availability

All relevant findings are presented descriptively in the paper.

## Supplementary material

For supplementary material accompanying this paper visit https://doi.org/10.1017/S0033291720002895. click here to view supplementary material
