# Supplementary material for: Autoantibody-associated psychiatric syndromes: a systematic literature review resulting in 145 cases
Source: Psychol Med. 2020 Sep 7;52(6):1135–46. doi: 10.1017/S0033291720002895 (PMC9069350; doi:10.1017/S0033291720002895)
Supplement: Supplementary file 1 [file S0033291720002895sup001.docx]

**Supplemental material**

|  |
| --- |
| exp Autoantibodies/ OR autoantibod*.mp. OR exp Antibodies/ OR. antibod*.mp. |
| **AND** |
| exp Encephalitis/ OR enceph*.mp. OR brain inflammation.mp. |
| **AND** |
| exp Receptors, AMPA/ OR (ampa1* or ampa2* or ampa-1* or ampa-2* or ampa 1* or ampa 2* or alfa-amino-3-hydroxy-5-methyl-4-isoxazolepropionic acid 1*).mp. OR exp Receptors, GABA-B/ OR (gaba-b* or gaba b* or gabab* or gamma-aminobutyric acid b* or gamma-aminobutyric acid-b*).mp OR exp Receptors, Dopamine D2/ OR dopamine D2*.mp. OR (dipeptidyl-peptidase-like protein 6* or DPPX*).mp. OR glycine receptor*.mp. OR exp Receptors, Metabotropic Glutamate/ OR (receptors, metabotropic glutamate* or mGluR1* or mGluR5*).mp. OR exp Receptors, Nicotinic/ OR (Receptors, Nicotinic* or acetylcholine receptors, nicotinic* or nACh-receptor* or nACh-R* or nACh receptor*).mp. OR (ANNA* or anti-neuronal nuclear*).mp. OR exp Receptors, N-Methyl-D-Aspartate/ OR (Receptors, N-Methyl-D-Aspartate* or receptors, n-methylaspartate* or NMDA* or NMDAR* or NMDA-R*).mp. OR exp Calcium Channels/ OR (vdcc* or voltage dependent calcium channels* or voltage-dependent calcium channels* or vgcc*).mp. OR exp Potassium Channels, Voltage-Gated/ OR (k+ channels, voltage-gated* or kv potassium channels* or Potassium Channels, Voltage- Gated* or LGI1* or Caspr2* or VGKC*).mp. OR Amphiphysin*.mp. OR (Adenylate Kinase 5* or Adenylate-Kinase 5* or AK5*).mp. OR (Collapsin Response Mediator Protein 5* or CRMP5* or CRMP-5* or CRMP 5*).mp. OR exp Glutamate Decarboxylase/ OR (glutamic acid decarboxylase 65* or GAD65* or GAD 65* or GAD-65* or acid decarboxylase 65, glutamic*).mp. OR (hu* or anti-hu*).mp. OR (Anti-Ma 2* or Ma 2* or Ma-2* or Anti-Ma1* or Ma 1* or Ma-1*).mp. OR (Homer 3* or Homer-3* or Homer3*).mp. OR (Purkinje cell cytoplasmic* or PCA-2* or PCA 2* or PCA2* or PCA-1* or PCA 1* or PCA1* or Yo* or anti-Yo*).mp. OR (Ri* or anti-Ri*).mp. OR (SRY-related HMG-box 1* or SOX1* or SOX-1* or SOX 1*).mp. OR (Tr* or anti-Tr*).mp. OR (Hashimoto's encephal* or Hashimoto encephal* or SREAT* or Steroid-responsive encephalopathy associated with autoimmune thyroiditis* or Steroid responsive encephalopathy associated with autoimmune thyroiditis* or steroid responsive encephal* or steroid-responsive encephal*).mp. OR (thyroid Peroxidase* or thyroidperoxidase* or thyroid-peroxidase* or antithyroid peroxidase*or anti-thyroid peroxidase* or anti-thyroidperoxidase* or anti-thyroid-peroxidase* or antithyroid-peroxidase* or anti thyroid peroxidase* or anti thyroidperoxidase* or anti-TPO* or anti TPO* or TPO*).mp. OR (thyroglobulin* or TG* or anti-thyroglobulin* or antithyroglobulin* or anti-TG* or anti TG*).mp. OR (thyrotropin receptor*, tsh receptor* or thyroid stimulating hormone receptor* or anti-thyrotropin* or antithyrotropin* or anti thyrotropin* or anti-TSH* or anti TSH* or TRAK*).mp. OR exp Aquaporin 4/ OR (aqp4* or aqp-4* or aqp 4* or aquaporin 4* or aquaporin-4*).mp. OR exp Myelin-Oligodendrocyte Glycoprotein/ OR (Myelin-Oligodendrocyte Glycoprotein* or Myelin Oligodendrocyte Glycoprotein* or MOG*).mp. OR (Zic4* or Zic 4* or Zic-4*).mp. OR IgLON5*.mp. OR (ARHGAP26* or GTPase activating protein 26).mp. |
| **AND** |
| exp Psychotic Disorders/ OR (psychotic* or schizo* or delusion* or catatonic* or catatonia* or psychos* or paranoid* or paranoia* or depersonalization* or derealization*).mp. OR exp Depressive Disorder/ OR exp Bipolar Disorder/ OR (Bipolar Disorder* or bipolar psych* or manic* or mania*).mp. OR exp Obsessive-Compulsive Disorder/ OR (Obsessive-Compulsive* or OCD* or anankastic*).mp. OR exp Anxiety Disorders/ OR anxiety*.mp. OR exp Delirium/ OR Delirium*.mp. OR exp Dementia/ OR (Dementia* or amentia*).mp. OR OR exp Cognition Disorders/ OR (cognition disorder* or MCI* or cognitive impairment* or cognitive dysfunction* or cognitive-decline* or mental deterioration*).mp. OR (dysexecutive* or executive dysfunction*).mp. OR exp Mood Disorders/ OR (affective* or mood* or depressive* or depression* or melancholia* or dysphoric*).mp. OR exp Depressive Disorder, Major/ OR exp Depression/ OR exp Depressive Disorder, Treatment-Resistant/ OR exp Affective Disorders, Psychotic/ OR exp Attention Deficit Disorder with Hyperactivity/ OR (ADHD* or ADDH* or ADD* or Attention Deficit* or Hyperactiv* or Hyperkinetic* or Minimal Brain Dysfunction* or MBD*).mp. OR exp Tic Disorders/ OR exp Tourette Syndrome/ OR (tic-disorder* or Tourette*).mp. OR exp Autistic Disorder/ OR (Autism* or autistic*).mp OR exp "Sleep Initiation and Maintenance Disorders"/ OR (("Sleep Initiation and Maintenance Disorders*") or insomnia*).mp. OR exp Mental Disorders/ OR (mental disorder* or psychiatric* or behavioral disorder*).mp. OR exp Paranoid Disorders/ OR exp Schizophrenia/ OR exp Schizophrenia, Catatonic/ OR exp Schizophrenia, Disorganized/ OR exp Schizophrenia, Paranoid/ OR exp Shared Paranoid Disorder/ OR exp Delusional Parasitosis/ OR exp Schizophrenia, Childhood/ |

**Supplemental Table 1: Search strategy.** Abbreviations: mp = title, abstract, original title, name of substance word, subject heading word, keyword heading word, protocol supplementary concept word, rare disease supplementary concept word, unique identifier, synonyms.

|  | **Patients with anti-NMDA-R antibodies** | **Patients with anti-VGKC antibodies** | **Patients with anti-AMPA-R antibodies** | **Other antineuronal antibodies *** |
| --- | --- | --- | --- | --- |
| **Number of cases** (% per subgroup of patients with antibodies against cell surface antigens) | 46 (58%) | 18 (23%) | 12 (15%) | 4 (5%) |
| **Females (%):**  **Males (%)** | 26 (57%):  20 (43%) | 10 (56%):  8 (44%) | 10 (83%):  2 (17%) | 3 (75%):  1 (25%) |
| **Age ± SD in years (range)** | 29.45 ± 16.41 (from 2 to 71) | 53.22 ± 24.39 (from 7 to 91) | 59.8 ± 14.03 (from 35 to 81) | 56.25 ± 11.09 (from 42 to 69) |
| **Antibody subtypes** (% per subgroup)* | Glu NR1: 8 (17%)  Isolated Glu NR2: 5 (11%)  Not reported: 33 (72%) | LGI1: 4 (22%)  CASPR2: 0 (0%)  Differentiation not performed/ reported: 14 (78%) |  | AQP4: 1 (25%)  DPPX: 1 (25%)  GABA_A_: 1 (25%)  Unknown: 1 (25%) |
| **Antibody investigation in serum/CSF (reported)** | Serum: 36 (78%)  CSF: 34 (74%) | Serum: 18 (100%)  CSF: 3 (17%) | Serum: 12 (100%)  CSF: 7 (58%) | Serum: 4 (100%)  CSF: 3 (75%) |
| **Antibody detection in serum/CSF** | Serum: 30 from 34 (88%)  CSF: 31 from 34 (91%)  Unclear: 0 (0%) | Serum: 18 from 18 (100%)  CSF: 3 from 3 (100%)  Unclear: 0 (0%) | Serum: 6 from 12 (50%)  CSF: 1 from 7 (14%)  Unclear: 6 (50%) | Serum: 3 from 4 (75%)  CSF: 3 from 3 (100%)  Unclear: 0 (0%) |
| **Syndromes** (% per subgroup)  - Schizophreniform  - Amnestic/dementia-like  - Confusional  - Depressive  - Manic  - Tic  - Sleep-disturbance  - Autistic  - Anxiety-compulsive | 26 (57%)  4 (9%)  7 (15%)  4 (9%)  3 (7%)  0 (0%)  0 (0%)  2 (4%)  0 (0%) | 2 (11%)  13 (72%)  1 (6%)  1 (6%)  0 (0%)  0 (0%)  1 (6%)  0 (0%)  0 (0%) | 0 (0%)  9 (75%)  3 (25%)  (0%)  (0%)  (0%)  (0%)  (0%)  (0%) | 1 (25%)  2 (50%)  0 (0%)  0 (0%)  0 (0%)  0 (0%)  1 (25%)  0 (0%)  0 (0%) |
| **Slight neurological symptoms** | 18 (39%) | 7 (39%) | 3 (25%) | 1 (25%) |
| **CSF overall alterations** (% of cases with reported CSF results) | 38 (from 42; 90%) | 10 (from 14; 71%) | 12 (from 12; 100%) | 4 (from 4; 100%) |
| **EEG overall alterations** (% of cases with reported EEG results) | 18 (from 30; 60%) | 7 (from 12; 58%) | 9 (from 11; 82%) | 1 (from 2; 50%) |
| **MRI overall alterations** (% of cases with reported MRI results) | 19 (from 43; 44%) | 10 (from 18; 56%) | 9 (from 12; 75%) | 3 (from 4; 75%) |
| **Tumor association overall** (% per full group) | 13 (28%) | 3 (17%) | 8 (67%) | 1 (25%) |
| **Improvement through immunomodulatory drugs in general** | 37 (from 40 treated patients; 93%) | 13 (from 15 treated patients; 87%) | 10 (from 11 treated patients; 91%) | 4 (from 4 treated patients; 100%) |

**Supplemental Table 2: Findings in patients with established antineuronal antibodies.** Abbreviations: CSF, cerebrospinal fluid; EEG, electroencephalography, MRI, magnetic resonance imaging.

|  | **Patients with exclusively psychiatric/ neurocognitive syndromes** |
| --- | --- |
| **Number of cases** (% per subgroup) | 99 from 145 included patients (68%) |
| **Females (%): Males (%)** | 65 (66%): 34 (34%) |
| **Age ± SD in years (range)** | 45.62 ± 22.57 (from 6 to 91 years) |
| **Antibody subtypes** (% per subgroup)* | Abs against cell surface antigens: 51 (52%)   - Anti-NMDA-R: 28 (55%) - Anti-VGKC: 11 (22%) **   - LGI1: 3 (6%)   - Anti-AMPA: 9 (18%) - Anti-GABA_A_: 1 (2%) - Anti-DPPX: 1 (2%) - Unknown: 1 (2%)   Abs against intracellular antigens: 13 (13%)   - Anti-GAD65: 1 (7%) - Anti-Ma1/2: 3 (23%) - Anti-Ri: 2 (15%) - Anti-Hu: 1 (7%) - Anti-AK5: 5 (38%) - Anti-BRSK2: 1 (7%)   Hashimoto encephalopathy/SREAT: 35 (35%)   - Anti-TPO: 14 (40%) - Anti-TG: 1 (3%) - Anti-TPO+TG: 19 (54%) |
| **Antibody investigation in serum/CSF** | Serum: 95 (96%)/ CSF: 53 (54%) |
| **Antibody detection in serum/CSF** | Serum: 80 from 95 (84%)/CSF: 37 from 53 (70%)/Unclear: 6 (6%) |
| **Syndromes** (% per subgroup) | Schizophreniform: 29 (29%)  Amnestic/dementia-like: 48 (48%)  Confusional: 10 (10%)  Depressive: 5 (5%)  Manic: 5 (5%)  Tic: 1 (1%)  Sleep-disturbance: 0 (0%)  Autistic: 0 (0%)  Anxiety-compulsive: 1 (1%) |
| **CSF overall alterations** (% of cases with reported CSF results) | 67 (from 86; 78%) |
| **EEG overall alterations** (% of cases with reported EEG results) | 42 (from 69; 61%) |
| **MRI overall alterations** (% of cases with reported MRI results) | 52 (from 94; 55%) |
| **Tumor association overall** (% per full group) | 21 (from 99; 21%) |
| **Improvement through immunomodulatory drugs in general** | 79 (from 85; 93%) |

**Supplemental Table 3: Clinical and additional findings of patients with isolated psychiatric or neurocognitive syndromes.** Abbreviations: CSF, cerebrospinal fluid; EEG, electroencephalography, MRI, magnetic resonance imaging.
